# Neoplasm Risk in Rheumatic Diseases Has No Correlation With Conventional Synthetic Disease-Modifying Anti-rheumatic Drugs Usage—A Population-Based Nested Case–Control Study

**DOI:** 10.3389/fmed.2020.00473

**Published:** 2020-08-26

**Authors:** Shaozhe Cai, Wuu-Tsun Perng, Jing Y. Huang, Jeng-Yuan Chiou, Lingli Dong, James C. Wei

**Affiliations:** ^1^Department of Rheumatology and Immunology, Tongji Hospital, Huazhong University of Science and Technology, Wuhan, China; ^2^Department of Recreational Sport and Health Promotion, National Pingtung University of Science and Technology, Neipu, Taiwan; ^3^Center for Health Data Science, Chung Shan Medical University Hospital, Taichung, Taiwan; ^4^Institute of Medicine, Chung Shan Medical University, Taichung, Taiwan; ^5^School of Health Policy and Management, Chung Shan Medical University, Taichung, Taiwan; ^6^Department of Medicine, Chung Shan Medical University Hospital, Taichung, Taiwan; ^7^Graduate Institute of Integrated Medicine, China Medical University, Taichung, Taiwan

**Keywords:** neoplasm risks, rheumatic diseases, nested case-control study, population based, disease-modyfying anti-rheumatic drugs

## Abstract

**Objectives:** To investigate whether there is an elevated neoplasm risk in patients with rheumatic diseases treated with conventional synthetic disease-modifying antirheumatic drugs (csDMARDs).

**Methods:** A population-based nested case–control study was performed by retrieving all patients newly diagnosed with rheumatoid arthritis (RA), systemic lupus erythematosus (SLE), and psoriatic arthritis (PsA) or psoriasis vulgaris (PsO) from the 2000 Longitudinal Health Insurance Database (LHID 2000) in Taiwan. Two hundred and sixty-one patients with neoplasm from 1997 to 2013 were enrolled in this study, and controls were matched in a 1:1 ratio with age, sex, and year of enrollment. Composition of demographic indices, comorbidities, medication usage, and differences in days of prescription of different medications between neoplasm and neoplasm-free (control) groups were compared.

**Results:** Between the control and neoplasm groups, no differences in ratio were observed in the usage of hydroxychloroquine (50.96 vs. 49.04%, *p* = 0.6616), methotrexate (26.82 vs. 27.59%, *p* = 0.8441), azathioprine (3.45 vs. 3.07%, *p* = 0.8052), and cyclophosphamide (1.15 vs. 2.30%, *p* = 0.3131) from enrollment to index date. Medications within 3 years before the index date in patients that had ≥3 months of comparable duration also showed no difference (hydroxychloroquine: 33.06 vs. 30.25%, *p* = 0.6404; methotrexate: 20.66 vs. 25.21%, *p* = 0.4018; azathioprine: 2.48 vs. 2.52%, *p* = 0.9835; cyclophosphamide: 0.83 vs. 0.84%, *p* = 0.9906). We also made a subgroup analysis focusing on RA and SLE patients; no difference between control and neoplasm group in both the ratio of usage and days of prescription of hydroxychloroquine, methotrexate, azathioprine, and cyclophosphamide was observed.

**Conclusion:** Neoplasm risk in patients with rheumatic diseases has no correlation with csDMARD usage.

## Introduction

Disease-modifying antirheumatic drugs (DMARDs) are a group of drugs defined by their effects of slowing down disease progression, which can be divided into biologic DMARDs and targeted synthetic DMARDs (tsDMARDs) and conventional synthetic DMARDs (csDMARDs). As conventional antirheumatic agents, csDMARDs are widely used for their efficacy and low cost. Hydroxychloroquine (HCQ), methotrexate (MTX), azathioprine (AZA), and cyclophosphamide (CTX) are commonly prescribed csDMARDs, particularly for systemic lupus erythematosus (SLE), rheumatoid arthritis (RA), and psoriasis (PsO) or psoriatic arthritis (PsA) patients, of which the usages have also been suggested in treatment guidelines of corresponding diseases (1–3). However, all these csDMARDs function as non-targeted immunosuppressive/modulatory agents, and some of the agents described above work via the interfering cell cycle (4–6). The immunosuppressive effects and the mechanism of action of these csDMARDs all indicate their potential carcinogenetic ability.

In consideration of the relative long-term usage of csDMARDs of patients with rheumatic diseases (RD), and the relationship of predisposition of malignancy to races and regions, based on a population-based database of health insurance research in Taiwan, we aimed here to compare the medication differences between RD patients without malignancy and RD patients with malignancy after exposure to csDMARDs targeting East Asians.

## Methods

### Study Design

We conducted a population-based nested case–control study by retrieving all patients newly diagnosed with RA, SLE, and PsA or PsO from the 2000 Longitudinal Health Insurance Database (LHID 2000) in Taiwan. This study was approved by the Ethics Review Board of Chung Shan Medical University. Patient informed consent was not required, as the NHIRD data files contain only de-identified secondary data.

### Study Base and Population

The data used in this study came from LHID 2000, a subset of Taiwan's National Health Insurance Research Database (NHIRD). The NHIRD database consists of all inpatient and outpatient visits, procedure codes, catastrophic illness files, and drug prescription data of the 23.5 million insured residents, whereas LHID 2000 contains all the original claim data for one million beneficiaries randomly sampled from the 2000 Registry for Beneficiaries of the National Health Insurance program. In the LHID 2000 database, the diagnosis and medication of patients are recorded via the diagnostic codes in the format of the International Classification of Diseases, Revision 9 (ICD-9), and medication code in the format of Anatomical Therapeutic Chemical (ATC) code, respectively.

All patients diagnosed with RA (ICD-9 code: 714.0), SLE (ICD-9 code: 710.0), and PsA (ICD-9 code: 696.0) or PsO (ICD-9 code: 696.1) from 1997 to 2013 were retrieved from LHID 2000, and their usage of hydroxychloroquine (ATC code: P01BA02), methotrexate (ATC code: L04AX03), azathioprine (ATC code: L04AX01), and cyclophosphamide (ATC code: L01AA01) was retrieved at the same time.

### Definition of Case and Control

Patients from 1997 to 2013 who had ever diagnosed with rheumatic diseases (RDs, include SLE, RA, PsA, and PsO) by rheumatologists were included (*n* = 8219). The criteria of ≥3 outpatient visits within 1 year and at least one admission were used to improve the validity of diagnosis. In order to ensure that all enrolled cases were newly diagnosed with RDs, which meant no previous diagnosis of any rheumatic diseases before, we excluded the cases, whose first-time diagnosis of RDs happened between 1997 and 2001 (*n* = 2379). The date of enrollment was the first date of visit for rheumatic diseases.

The selected patients were divided into neoplasm group and neoplasm-free (control) group, respectively. The patients, who had previous diagnosis of neoplasm (ICD9 codes: 140-208) in the neoplasm group, were also excluded (*n* = 432). We created a matched sample by matching the remaining subjects in neoplasm-group subjects to the subjects in the neoplasm-free group by age at the index date (the last day of 2013 for neoplasm-free patients and the first day of neoplasm confirmation for neoplasm patients) and gender. Finally, we realized a 1:1 match (neoplasm group, *n* = 261; control group, *n* = 261).

### Confounders

Comorbidities were potential confounders and were identified with ICD-9 codes. Thyroid disorders (ICD-9 codes: 240, 241, 242, 244.9, 245.0, 245.1, 245.2), viral hepatitis (ICD-9 code: 070), infectious mononucleosis (ICD-9 code: 075), hypertension (ICD-9 codes: 401–405), diabetes mellitus (ICD-9 code: 250), hyperlipidemia (ICD-9 code: 272), coronary artery disease (ICD-9 codes: 410–414), CKD (ICD-9 code: 585), asthma (ICD-9 code: 493), COPD (ICD-9 codes: 490–496), esophageal disease (ICD-9 codes: 530.0–530.9), gastrointestinal ulcer (ICD-9 codes: 531–534), regional enteritis (including Crohn's disease) and ulcerative colitis (ICD-9 code: 556), and chronic liver disease (ICD-9 code: 571) were identified as comorbidities in this study.

### Statistical Analysis

Composition of characteristic indices, comorbidities, and medication between neoplasm and neoplasm-free (control) groups were compared with Chi-square tests. Differences of days of prescription of different medications were presented as Median ± Interquartile range (IQR) of each group and compared using the Wilcoxon rank-sum test. A two-tailed *p* < 0.05 was considered statistically significant. All statistical analyses were conducted using SAS Statistics software (version 9.4; SAS Institute, Inc., Cary, NC, USA).

## Results

**Figure 1:**
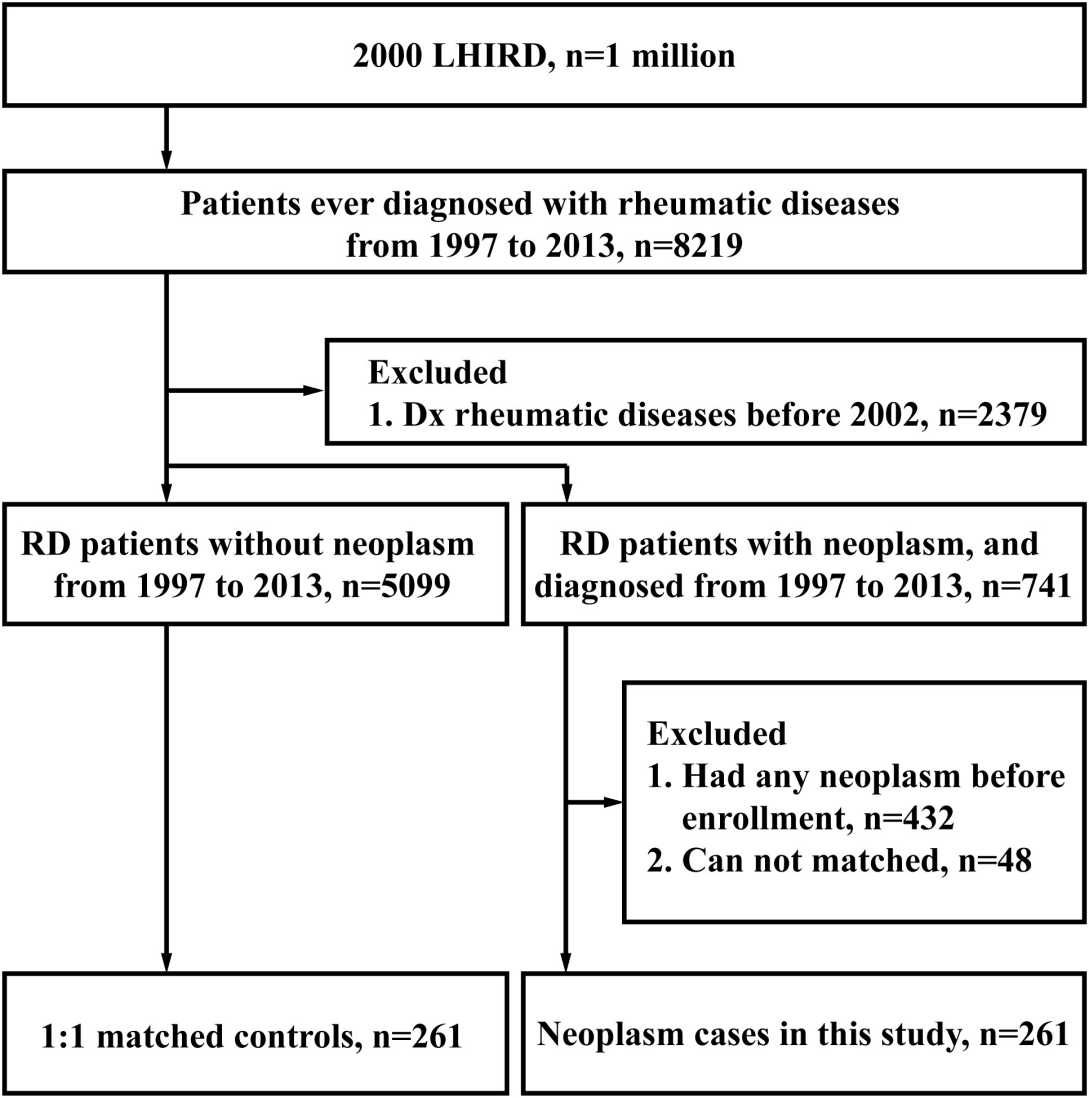
Study design and flow.

Between 1997 and 2013, 8,219 patients ever diagnosed with rheumatic diseases (RD) were selected from the 2000 Longitudinal Health Insurance Database (LHID 2000). Two thousand three hundred seventy-nine participants were excluded for their diagnosis before 2002; 5099 RD participants without neoplasms and 741 RD participants with neoplasms were remained. After exclusion of 432 participants with neoplasms diagnosed before enrollment, we made a 1:1 match for the neoplasm cases with neoplasm-free cases based on the criteria listed in Methods. Finally, both the neoplasm-free (control) group and the neoplasm group consisted of 261 cases, as shown in Figure 1.

The ratios of types of rheumatic diseases, age at index date, urbanization state, and length of hospital stay within 2 years before index date are similar between the neoplasm and control groups, as shown in Table 1. For the comorbidities, except that the viral hepatitis (6.71 vs. 15.71%) and chronic kidney disease (CKD, 4.21 vs. 9.20%) were more prevalent in the neoplasm group, ratios of other comorbidities listed in Table 1 showed no statistical difference.

**Table 1:** Characteristics among groups.

	**Control**	**Neoplasm patients**	***p*-value**
Rheumatic diseases			0.1260
Only SLE	36 (13.79%)	43 (16.48%)	
Only RA	186 (71.26%)	165 (63.22%)	
Only PsA	3 (1.15%)	7 (2.68%)	
Only PsO	20 (7.66%)	21 (8.05%)	
Combined 2 diseases	14 (5.36%)	25 (9.58%)	
Combined ≥3 diseases	2 (0.77%)	0 (0.00%)	
Age at index date			1.0000
<60	129 (49.43%)	129 (49.43%)	
≥60	132 (50.57%)	132 (50.57%)	
Sex			1.0000
Female	184 (70.50%)	184 (70.50%)	
Male	77 (29.50%)	77 (29.50%)	
Urbanization			0.368
Urban	156 (59.77%)	166 (63.60%)	
Suburban	105 (40.23%)	95 (36.40%)	
Length of hospital stay within 2 years before index date			0.1482
0	171 (65.52%)	146 (55.94%)	
1–6	33 (12.64%)	39 (14.94%)	
7–13	26 (9.96%)	32 (12.26%)	
≥14	31 (11.88%)	44 (16.86%)	
Comorbidities (within 2 year before index date)			
Thyroid disorders	14 (5.36%)	25 (9.58%)	0.0671
Viral hepatitis	17 (6.51%)	41 (15.71%)	0.0008
Infectious mononucleosis	0 (0%)	0 (0%)	
Hypertension	107 (41.00%)	125 (47.89%)	0.1129
Diabetes mellitus	48 (18.39%)	69 (26.44%)	0.0275
Hyperlipidemia	62 (23.75%)	74 (28.35%)	0.2315
Coronary artery disease	50 (19.16%)	51 (19.54%)	0.9118
CKD	11 (4.21%)	24 (9.20%)	0.0229
Asthma	24 (9.2%)	28 (10.73%)	0.5588
COPD	44 (16.86%)	54 (20.69%)	0.2624
Esophageal disease	46 (17.62%)	49 (18.77%)	0.7336
Gastrointestinal ulcer	86 (32.95%)	100 (38.31%)	0.2007
Regional enteritis and ulcerative colitis	0 (0%)	0 (0%)	
Chronic liver disease	51 (19.54%)	69 (26.44%)	0.0611

Similar comparable durations of the control and neoplasm groups are shown with an interquartile range in Table 2. Based on the similarity of time intervals of comparable duration, we compared the usage of HCQ, MTX, AZA, and CTX between these two groups. No differences in ratio were observed in the usage of hydroxychloroquine (50.96 vs. 49.04%, *p* = 0.6616), methotrexate (26.82 vs. 27.59%, *p* = 0.8441), azathioprine (3.45 vs. 3.07%, *p* = 0.8052), and cyclophosphamide (1.15 vs. 2.30%, *p* = 0.3131) (Table 3.1). In order to investigate the more precise carcinogenetic influences related to medication, we also compared the drug usages within 3 years before the index date (for neoplasm patients, the date of neoplasm diagnosis). No difference was also observed between the control group and neoplasm groups (Table 3.2). The similarities were also observed in the days of prescription of these medications between the control group and neoplasm group. Considering that a large part of cases enrolled in our study were RA or SLE patients, we also made a subgroup analysis focusing on these cases: In both RA and SLE patients, there was no difference between control and neoplasm groups, in both the ratio of usage and days of prescription of these nbDMARDs (Table 4).

**Table 2:** Time interval (month) of comparable duration.

	**Control**	**Neoplasm-free patients**
Time interval		
Min	0	0
Q1	12	12
Median	32	32
Q3	62	62
Max	137	136

**Table 3.1:** Medication between groups from enrollment to index date.

	**Control**	**Cancer patients**	***p*-value**
Hydroxychloroquine	133 (50.96%)	128 (49.04%)	0.6616
Days of prescription	189 ± 613	250 ± 700	0.6393
Methotrexate	70 (26.82%)	72 (27.59%)	0.8441
Days of prescription	297.5 ± 777	332.5 ± 562.5	0.6630
Azathioprine	9 (3.45%)	8 (3.07%)	0.8052
Days of prescription	154 ± 448	234.5 ± 1743	0.7052
Cyclophosphamide	3 (1.15%)	6 (2.30%)	0.3131
Days of prescription	935 ± 910	212 ± 291	0.7086

**Table 3.2:** Medication between groups within 3 years.

	**Control *n* = 121**	**Cancer patients *n* = 119**	***p*-value**
Hydroxychloroquine	40 (33.06%)	36 (30.25%)	0.6404
Days of prescription	479 ± 848.5	588 ± 644	0.5108
Methotrexate	25 (20.66%)	30 (25.21%)	0.4018
Days of prescription	592 ± 714	399 ± 770	0.5850
Azathioprine	3 (2.48%)	3 (2.52%)	0.9835
Days of prescription	532 ± 588	791 ± 499	0.4227
Cyclophosphamide	1 (0.83%)	1 (0.84%)	0.9906
Days of prescription	448 ± 0	182 ± 0	–

**Table 4:** Subgroup analysis for medication among groups from enrollment to index date.

	**Control**	**Cancer patients**	***p*-value**
**In SLE-only patients**	***n*** **=** **36**	***n*** **=** **43**	
Hydroxychloroquine	19 (52.78%)	20 (46.51%)	0.5790
Days of prescription	231 ± 715	141 ± 636	0.9332
Methotrexate	2 (5.56%)	2 (4.65%)	0.8551
Days of prescription	525 ± 434	175 ± 210	0.3293
Azathioprine	2 (5.56%)	3 (6.98%)	0.7961
Days of prescription	1,215 ± 2,262	2,219 ± 2,207	0.7872
Cyclophosphamide	2 (5.56%)	4 (9.30%)	0.5313
Days of prescription	518 ± 910	211.5 ± 642	1.0000
**In RA-only patients**	***n*** **=** **186**	***n*** **=** **165**	
Hydroxychloroquine	105 (56.45%)	93 (56.36%)	0.9868
Days of prescription	182 ± 536	287 ± 700	0.3733
Methotrexate	51 (27.42%)	53 (32.12%)	0.3356
Days of prescription	335 ± 861	385 ± 679	0.3496
Azathioprine	4 (2.15%)	2 (1.21%)	0.4984
Days of prescription	476 ± 364	91 ± 154	0.2994
Cyclophosphamide	1 (0.54%)	1 (0.61%)	0.9323
Days of prescription	935 ± 0	214 ± 0	–

## Discussion

According to our knowledge, this is the first population-based, nested case–control study targeting East Asians, which investigated the carcinogenetic effects of commonly used csDMARDs (including HCQ, MTX, AZA, and CTX) on patients suffering from rheumatic diseases. For this purpose, we compare the difference of ratio and medication time (via days of prescription) in the usage of different csDMARDs, which was based on a 1:1 match between neoplasm and neoplasm-free patients. Our study showed no differences in the indexes described above, which meant the usage of these four nbDMARDs had no correlation with carcinogenesis.

As the backbone of treatment to rheumatic diseases, csDMARDs can help attenuate the disease activity and slow down the progression, while avoiding the severe side effects resulting from long-term usage of steroids in high dose. The relative lower cost of csDMARDs also makes them easier to be accepted by most patients. According to the mechanism of HCQ, MTX, AZA, and CTX, the actions resulting from these csDMARDs (including immunosuppression, cytotoxicity, and etc.) have the potential to contribute to the pathogenesis of neoplasm with long-term usage (4–7). Therefore, the potential long-term neoplasm risk prompts us to investigate whether there is any relationship between carcinogenesis and usage of some csDMARDs.

Several investigations had focused on the incidences of neoplasms in some RDs under exposure to csDMARDs. However, the conclusions from these studies were controversial. For RA, investigation from Jin et al. targeting 13,210 Chinese RA patients identified malignancy as one of the major comorbidities of RA with a prevalence of 0.6% at baseline, and MTX usage was negatively associated with malignancy (HR = 0.57, *p* = 0.02) (8), while Solomon et al. showed that cancer risk was elevated for methotrexate users compared with other nbDMARDs and TNF antagonists (9). For SLE, a nested case–control study based on a national Swedish systemic lupus erythematosus cohort showed a correlation of the elevation of lymphoma risk to presentation of hematological or sicca symptoms, or pulmonary involvement in SLE, but no correlation to cyclophosphamide or azathioprine usage (10), while a case–cohort analysis based on a multisite SLE cohort showed a suggestion of greater exposure to cyclophosphamide and to higher cumulative steroids in lymphoma cases than the cancer-free controls (11). For PsA, Fiorentino et al. showed that long-term treatment with methotrexate or ustekinumab was not associated with increased malignancy risk vs. no exposure (12), while the risk of squamous cell carcinoma and lymphoproliferative diseases was elevated under the exposure of MTX, as shown in several other researches (13).

As described above, several studies had observed an association between elevated risk of malignancy in some systems and some rheumatic diseases, like RA, SLE, and PsO. In a review of Klein et al., they indicated that the association between lymphoma risk and RA might be explained mainly by three theories: genetic predisposition, persistence of long-standing disease activity with continued immune stimulation, and the role of anti-RA therapy given (14, 15). The association between neoplasm risk and other RDs can also be roughly conducted as these three reasons. Beside the controversial viewpoints, investigations mentioned above targeted mainly Europeans and Americans and always overlooked some confounders that could influence patients' long-term neoplasm risk. In our study, study subjects were confined to East Asians (different genetic background from people from other areas of the world), and disease severities (presented with length of hospital stay within 2 years before index date) were similar between the neoplasm group and control group, which helped reduce confounders resulting from genetic predisposition and variation of disease activity of enrolled cases.

Our study had also limitations. Firstly, this study was based on a claim-based health insurance database, and although the length of hospital stay of cases was well-documented, and also regarded as a marker for disease severity in this study, we could not evaluate their activity and severity in details, which may introduce the bias into the final results. Secondly, contribution of lifestyle to cancers, such as smoking and alcohol, is lacking in our database. However, we did match life style-related diseases such as diabetes, COPD, and liver disease to reduce the confounding. We also excluded patients with prior cancer history to enhance baseline comparability between cases and controls. Thirdly, it is difficult to ascertain from “days of prescription” the duration of exposure and adherence to treatment directly (or very precisely), but the days of prescription can reflect the requirements of specific medication of patients, which can indicate the real usage of that medication indirectly. Lastly, although this is a nested case–control study, and the compositions of cases of different diseases were similar between different groups after matching, the composition of different diseases varied significantly (from around 1 to 70%). Therefore, clinical trials with a higher level and larger scale should be made in the future, to give a more precise and detailed answer to this important problem in clinical practice, and basic researches should also be made to investigate the precise carcinogenetic effects of these csDMARDs in rheumatic patients at the level of mechanism.

In summary, we compared the medication differences of some commonly used csDMARDs between rheumatic diseases patients with and without neoplasms, which showed no differences and indicated no correlation between csDMARD usage and neoplasm risk in patients with rheumatic diseases.

## Data Availability Statement

The raw data supporting the conclusions of this article will be available upon reviewers' request. Requests to access the datasets should be directed to jccwei@gmail.com.

## Ethics Statement

This study was approved by the Ethics Review Board of Chung Shan Medical University.

## Author Contributions

JW, LD, and SC participated in the design of this study. SC wrote this manuscript. JH and J-YC made the statistical analyses. W-TP provided very useful advices. All authors contributed to the article and approved the submitted version.

## Conflict of Interest

The authors declare that the research was conducted in the absence of any commercial or financial relationships that could be construed as a potential conflict of interest.
